# Beyond the heart: a review exploring non-cardiovascular effects of vasoactive agents

**DOI:** 10.3389/fphar.2025.1533437

**Published:** 2025-07-10

**Authors:** Peng Lan, Lina Chen, Chen Zhang, Jun Ni, Peihao Yu, Jiancang Zhou

**Affiliations:** Department of Critical Care Medicine, Sir Run Run Shaw Hospital, Zhejiang University School of Medicine, Hangzhou, China

**Keywords:** vasoactive, norepinephrine, epinephrine, dopamine, vasopressin

## Abstract

Vasoactive agents, traditionally recognized for their roles in cardiovascular regulation, have garnered increasing attention for their non-cardiovascular effects across various physiological systems. This review explores the multifaceted roles of vasoactive agents such as catecholamines, vasopressin, and angiotensin II beyond their cardiovascular implications. We examine the mechanisms of action, focusing on receptor interactions and the implications for various physiological systems. Key areas of impact include the central nervous system, where vasoactive agents influence mood, cognition, and neurological function, alongside potential neurotoxicity. Additionally, we discuss gastrointestinal effects, including motility and secretion, as well as renal implications related to blood flow and acute kidney injury risk. The endocrine effects are also addressed, particularly regarding insulin and glucagon secretion. Furthermore, we analyze hematological effects on coagulation and endothelial function, emphasizing the risk factors for thromboembolic events. The clinical implications of this review underscore the importance of monitoring non-cardiovascular effects in patient management and developing strategies to mitigate associated risks. Future research should focus on unraveling the detailed mechanisms of vasoactive agent-receptor interactions and their resulting organ responses, to minimize complications arising from clinical use.

## 1 Introduction

Vasoactive drugs, including vasopressors and inotropes, are critical components in the management of various acute medical conditions, particularly those involving cardiovascular instability (Overgaard and Dzavík, 2008; Annane et al., 2018). These agents function primarily to enhance cardiac output (CO) or increase vascular tone, thereby improving tissue perfusion and oxygen delivery (Boerma and Ince, 2010). Commonly used vasoactive drugs include catecholamines like norepinephrine (NE) and epinephrine (EPI), as well as non-catecholamine agents such as vasopressin and phosphodiesterase (PDE) inhibitors.

Catecholamines, such as NE and EPI, primarily function by inducing vasoconstriction through α-adrenergic receptor activation, which increases systemic vascular resistance (SVR) and mean arterial pressure (MAP). NE is particularly effective in raising blood pressure while maintaining CO due to its mixed α1 and β1 activity (Hernández et al., 2019). Conversely, EPI exhibits a broader spectrum of action, affecting both α and β receptors to enhance heart rate and cardiac contractility while also promoting vasodilation at lower doses through β2 receptor activation (Johnson and Moskowitz, 2024). This dual action allows for nuanced management of hemodynamic status in critically ill patients. Inotropes like dobutamine and milrinone focus on enhancing cardiac contractility. Dobutamine predominantly stimulates β1 receptors, leading to increased CO with minimal impact on SVR (Franco et al., 2021). Milrinone, a PDE inhibitor, increases intracellular cyclic adenosine monophosphate (cAMP) levels, resulting in improved myocardial contractility and peripheral vasodilation (Gist et al., 2021). These agents are particularly valuable in patients with heart failure or low CO states.

**FIGURE 1:**
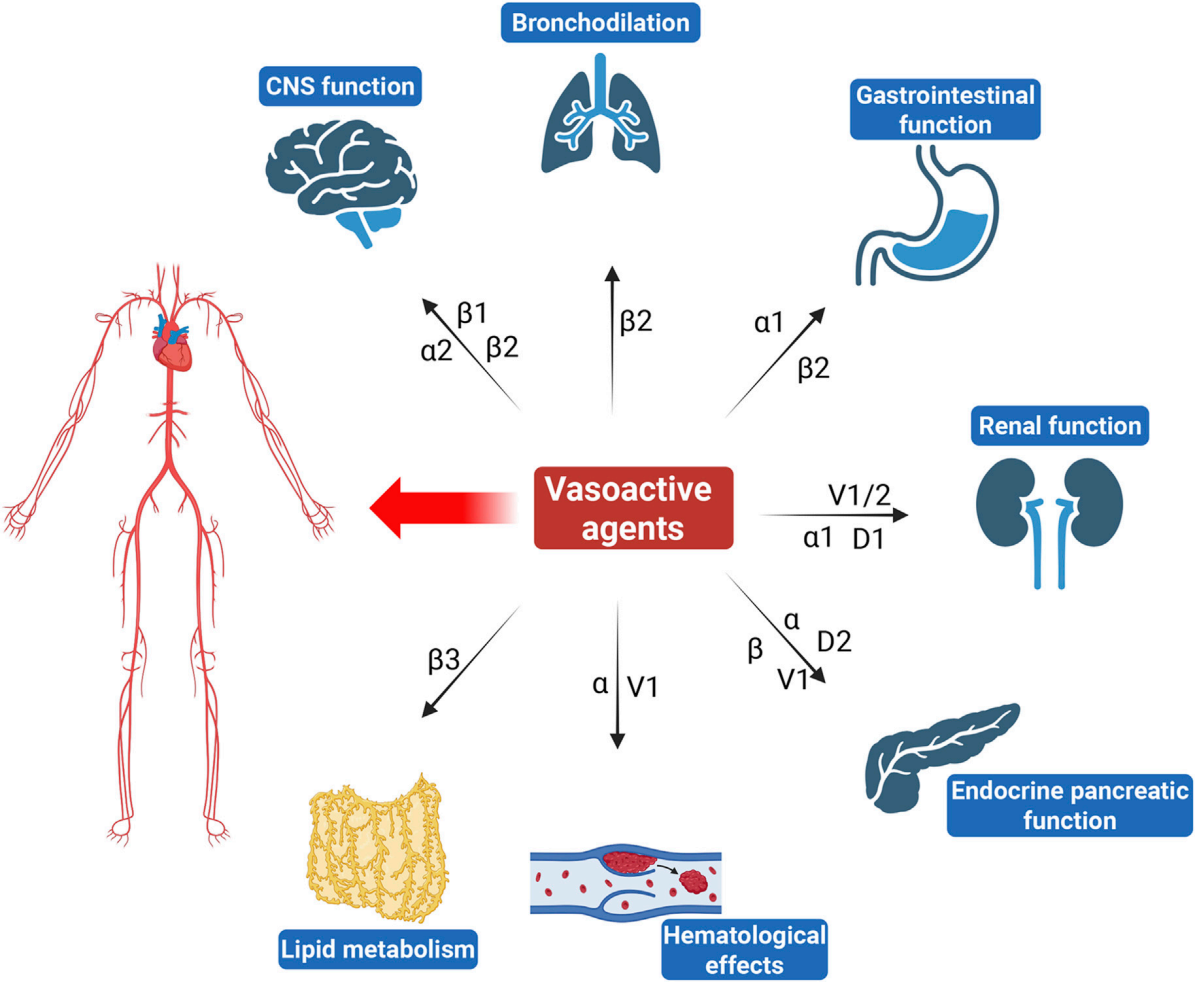
Overview of the effects of commonly used vasoactive agents on non-cardiovascular systems.

The complexity of vasoactive agents arises from their multifaceted mechanisms of action (Figure 1). They interact with various receptors, including adrenergic, dopaminergic, and vasopressin receptors, leading to diverse physiological responses (Shankar et al., 2022). For instance, while NE predominantly increases SVR and blood pressure through α1 receptor activation, it may also induce significant renal vasoconstriction, potentially compromising renal function. Vasopressin not only raises blood pressure but also influences renal function by promoting water reabsorption and can affect coagulation pathways through its action on V receptors (Demiselle et al., 2020). Similarly, dopamine exhibits dose-dependent effects that can lead to both renal vasodilation at low doses and vasoconstriction at higher doses, illustrating the delicate balance between therapeutic benefits and risks (Armando et al., 2011).

The importance of exploring the non-cardiovascular effects of vasoactive agents lies in their potential implications for patient management. Adverse effects such as digital ischemia from excessive vasoconstriction or altered microcirculation can impact organ perfusion and function (Woolsey and Coopersmith, 2006). Moreover, the interplay between vasoactive agents and other medications can further complicate clinical scenarios, necessitating a comprehensive understanding of these interactions (Gordon et al., 2014).

Thus, a thorough investigation into the non-cardiovascular effects of vasoactive agents is crucial for healthcare professionals involved in critical care settings. This review aims to elucidate these effects, providing insights that will enhance our understanding of how these medications can be used safely and effectively across various clinical contexts.

## 2 Mechanisms of action

### 2.1 Overview of vasoactive drug classes

Catecholamines, including NE, EPI, and dopamine, are pivotal in the management of various clinical conditions, particularly in acute settings such as shock and heart failure (Table 1). These agents exert their effects primarily through adrenergic receptors, which are G protein-coupled receptors that mediate a range of physiological responses (Xu et al., 2023). The mechanisms by which catecholamines exert their effects involve complex signaling pathways. Upon binding to their respective receptors, catecholamines activate adenylate cyclase via G proteins, increasing cAMP levels within the cell (Motiejunaite et al., 2021). This cascade leads to enhanced calcium influx through voltage-gated calcium channels and increased intracellular calcium concentrations, which are crucial for muscle contraction in cardiac and vascular tissues (Motiejunaite et al., 2021; Riccardi et al., 2024).

**TABLE 1:** Overview of vasoactive agents in clinical use.

Agent	Mechanism of action	Physiological effects	Clinical use	Cooperation
EPI	α1/β1/β2 agonist	↑HR, ↑CO, ↑BP, ↑SVR	Cardiac arrest, anaphylaxis	EPI potentiates NE in shock states. Cooperates with dobutamine to increase CO.
NE	α1 agonist (predominantly)	↑BP, ↑SVR	Septic shock	Cooperates with dopamine to increase renal perfusion at lower doses. Potentiates the effects of EPI in shock
Dopamine	Dose-dependent (Dopaminergic/α/β agonist)	↑HR, ↑CO, ↑BP, ↑SVR (high doses), renal vasodilation (low dose)	Heart failure, shock	Cooperates with NE to improve CO. EPI potentiates the effects in shock
Dobutamine	β1 agonist	↑CO, ↓SVR, ↑HR	Heart failure	Cooperates with vasopressors to improve CO
Phenylephrine	Pure α1 agonist	↑BP, ↑SVR, ↓HR, ↓CO	Hypotension	Cooperates with NE to raise SVR without affecting heart rate. Helps in reducing the need for catecholamines in cases of low vascular tone
Vasopressin	V1 receptor agonist	↑BP, ↑SVR	Vasodilatory shock	Cooperates with NE to improve MAP in shock, especially in septic shock
Milrinone	PDE inhibitor	↑CO, ↓BP, ↓SVR	Heart failure	Cooperates with vasopressors (like EPI) to improve CO
Angiotensin II	Angiotensin receptor agonist	↑BP, ↑SVR	Vasodilatory shock	Cooperates with NE and vasopressin in shock states to improve blood pressure and organ perfusion. AT1 and AT2 mediate opposing effects

PDE, phosphodiesterase; HR, heart rate; CO, cardiac output; SVR, systemic vascular resistance; NE, norepinephrine; EPI, epinephrine; BP, blood pressure.

NE is commonly used as a first-line vasopressor in septic shock (Evans et al., 2021), cardiogenic shock (Hu and Mathew, 2022) and acute hypotensive states. It primarily acts on α1 adrenergic receptors, leading to vasoconstriction and increased SVR, which enhances blood pressure (Motiejunaite et al., 2021; Perez, 2021). Additionally, NE stimulates β1 adrenergic receptors in the heart, resulting in increased myocardial contractility and heart rate (Motiejunaite et al., 2021). However, its effects on β2 adrenergic receptors are minimal, making it less effective for inducing vasodilation compared to other catecholamines like EPI. EPI exhibits dose-dependent effects mediated through its interaction with adrenergic receptors. It strongly activates β1 adrenergic receptors and moderately stimulates β2 and α1 adrenergic receptors (Evans et al., 2021). At lower doses, β1 receptor effects predominate, leading to heightened CO and reduced SVR, while MAP may fluctuate. In contrast, higher doses enhance α1 and β2 receptor activity, resulting in elevated SVR and further increases in CO (Evans et al., 2021). Adverse effects, such as cardiac arrhythmias and compromised splanchnic blood flow, are potential risks. Additionally, EPI stimulates β2 receptors in skeletal muscle, boosting aerobic lactate production (Evans et al., 2021). This effect complicates the interpretation of serum lactate levels as a marker for guiding resuscitation efforts. Dopamine also exerts dose-dependent effects by targeting dopamine-1 (D1), α1, and β1 adrenergic receptors. s (Elkayam et al., 2008; Armando et al., 2011). At lower doses, dopamine primarily activates D1 receptors, promoting vasodilation in vascular beds such as the renal circulations, though confer no significant protection from renal dysfunction. As doses escalate, α-adrenergic effects become dominant, inducing vasoconstriction and elevated SVR. Concurrent β1 adrenergic receptor stimulation at higher doses enhances cardiac activity but also raises the risk of dose-limiting arrhythmias. This dose-dependent nature allows for tailored therapeutic approaches depending on the clinical scenario (Dorn, 2010; Huang et al., 2016).

While catecholamines are vital in acute care settings for managing cardiovascular instability, their non-specific actions can lead to significant side effects. For instance, prolonged use of NE can cause peripheral ischemia due to excessive vasoconstriction (Daroca-Pérez and Carrascosa, 2017). Similarly, EPI’s broad effects can result in metabolic disturbances and increased myocardial oxygen consumption (Ducrocq et al., 2012). Therefore, careful titration and monitoring are essential when using these agents to balance therapeutic benefits with potential risks.

Non-catecholamine vasoactive agents, particularly vasopressin and PDE inhibitors, play crucial roles in the management of various clinical conditions, especially in the context of shock and heart failure. Vasopressin, also known as antidiuretic hormone, is synthesized in the hypothalamus and released from the posterior pituitary gland in response to increased plasma osmolality or decreased blood volume (Holmes et al., 2004). It primarily acts through three receptor subtypes: V1a, V1b, and V2 receptors. Activation of V1a receptors leads to vasoconstriction, thereby increasing SVR and blood pressure. This effect is particularly beneficial in states of hypotension, such as septic shock. Vasopressin can increase MAP without significantly affecting CO, making it a valuable adjunctive therapy in critically ill patients. However, its non-selective receptor activation can lead to side effects such as hyponatremia and potential procoagulant effects due to increased platelet aggregation (Demiselle et al., 2020).

PDE inhibitors are another class of non-catecholamine vasoactive agents that enhance CO and improve hemodynamics through different mechanisms. PDE inhibitors work by preventing the breakdown of cAMP and cyclic guanosine monophosphate (cGMP), which are critical second messengers involved in various physiological processes including heart contractions, smooth muscle relaxation in blood vessels and neuronal signaling (Newton et al., 2016). By increasing cAMP levels, these agents enhance myocardial contractility (positive inotropic effect) and promote vasodilation. Milrinone is particularly known for its ability to improve cardiac function in patients with heart failure by increasing contractility while also causing peripheral vasodilation (Mathew et al., 2021). The use of PDE inhibitors can be beneficial in patients with acute decompensated heart failure or cardiogenic shock. They can improve CO and reduce pulmonary congestion without significantly increasing heart rate or myocardial oxygen demand (Chi et al., 2013; Rieg et al., 2014). However, caution is warranted due to potential side effects such as hypotension and arrhythmias (Chong et al., 2018).

### 2.2 Receptor interactions

Adrenergic receptors, classified into α and β subtypes, are pivotal in mediating the physiological responses to catecholamines. These G protein-coupled receptors play critical roles in the sympathetic nervous system, orchestrating various bodily functions in response to stressors.

#### 2.2.1 α adrenergic receptors

There are two main types of α receptors: α1 and α2. α1 receptors are predominantly located on vascular smooth muscle. Activation of α1 receptors in the cardiovascular system by NE and EPI leads to vasoconstriction, increasing SVR and blood pressure. This mechanism is particularly important during the “fight or flight” response, where increased blood flow to essential organs including heart, brain, and skeletal muscles is necessary for survival (Borkar and Fadok, 2024). However, activation of α1 receptor in the bladder and gastrointestinal (GI) tract causes contraction of smooth muscles, inhibiting non-essential functions during stress (Michel and Vrydag, 2006). In contrast, α2 receptors primarily function as inhibitory autoreceptors located on presynaptic nerve terminals (Zhang et al., 2009). When activated, they decrease the release of NE, providing a negative feedback mechanism that modulates sympathetic activity. This action can lead to a reduction in blood pressure and heart rate when drugs like clonidine or dexmedetomidine are used, which selectively activate central α2 receptors to treat hypertension and manage anxiety (Pichot et al., 2012). Moreover, α2 receptor activation in the central nervous system (CNS) can produce sedation and analgesia, contributing to their role in pain modulation (Giovannitti et al., 2015).

#### 2.2.2 β adrenergic receptors

β adrenergic receptors are further subdivided into three types: β1, β2, and β3. β1 receptors are primarily found in the heart, mediating increases in heart rate (chronotropy) and myocardial contractility (inotropy) upon stimulation by catecholamines. This response enhances CO during stressful situations. Furthermore, β1 receptor activation in the kidneys stimulates renin release, leading to increased blood volume and pressure through the renin-angiotensin-aldosterone system (Sandilands and O'Shaughnessy, 2019). β2 receptors are predominantly located in smooth muscle tissues, including bronchioles and blood vessels. Activation of β2 receptors results in relaxation of smooth muscles, leading to bronchodilation and vasodilation (Kotlikoff and Kamm, 1996). This effect is crucial for improving airflow during respiratory distress and enhancing blood flow to skeletal muscles during physical exertion. Importantly, β2 receptor activation can counteract some of the vasoconstrictive effects mediated by α1 receptors in certain vascular beds (Wachter and Gilbert, 2012). While less commonly discussed, β3 receptors are involved in lipolysis in adipose tissue and may play a role in regulating energy metabolism (Cero et al., 2021). Their activation can lead to increased energy expenditure and thermogenesis.

The interplay between α and β adrenergic receptors allows for a finely tuned physiological response to stressors. During a fight-or-flight situation, α1-mediated vasoconstriction ensures that vital organs receive adequate blood flow while β2-mediated vasodilation enhances oxygen delivery to skeletal muscles. This balance is essential for optimizing performance under stress. Moreover, the distribution of these receptors varies across different tissues, allowing for localized responses tailored to specific physiological needs. While both α1 and β2 receptors may be present in a given tissue (e.g., blood vessels), their differential activation can result in opposing effects—vasoconstriction versus vasodilation—depending on the prevailing hormonal environment (Ahlquist, 1976; Richards et al., 2017).

#### 2.2.3 V receptors

Vasopressin acts primarily through three receptor subtypes: V1a, V1b, and V2 receptors. Activation of V1a receptors leads to vasoconstriction and also influences various non-cardiovascular functions, including enhancing platelet aggregation and promoting renal vasoconstriction (Honda and Takano, 2009). V2 receptors mediate the antidiuretic effects of vasopressin by promoting water reabsorption (Carty et al., 2024). This action is crucial for maintaining fluid balance and osmotic homeostasis. Additionally, V2 receptor activation may have implications for fluid retention in states of hypovolemia or dehydration (Lemmens-Gruber and Kamyar, 2006). V1b receptors are involved in stimulating adrenocorticotropic hormone (ACTH) release, which plays a role in stress responses. The activation of these receptors can influence cortisol secretion, thereby affecting metabolic processes and immune responses (Meijer et al., 2011).

#### 2.2.4 AT receptors

Angiotensin II (Ang II) is a potent vasoconstrictor primarily acting through two main receptor subtypes: AT1 and AT2 receptors. AT1 receptors mediate most of the well-known effects of Ang II, including vasoconstriction, increased blood pressure, and stimulation of aldosterone secretion from the adrenal cortex. The activation of AT1 receptors not only regulates blood pressure but also mediates non-cardiovascular effects such as promoting inflammation, fibrosis, and cellular hypertrophy in various tissues (Ruiz-Ortega et al., 2006). This pro-inflammatory action can contribute to the pathogenesis of conditions like hypertension and heart failure (Carter et al., 2024). In contrast to AT1 receptors, AT2 receptors generally mediate opposing effects, including vasodilation and inhibition of cell growth. They are involved in tissue repair processes and may exert protective effects against hypertrophy and fibrosis (Namsolleck et al., 2014). The balance between AT1 and AT2 receptor activation is crucial for maintaining cardiovascular homeostasis and influencing non-cardiovascular outcomes.

## 3 Non-cardiovascular effects

### 3.1 Effects on the CNS

#### 3.1.1 Effects on mood and cognition

Catecholamines are well-known for their role in the “fight or flight” response, where they prepare the body for stressful situations. NE, in particular, has been implicated in mood regulation and cognitive functions. It is a key neurotransmitter in the brain’s arousal system and is associated with attention, learning, and memory (Maletic et al., 2017). Dysregulation of NE levels has been linked to mood disorders such as depression and anxiety. For example, increased NE activity is often observed in states of heightened stress or anxiety, while decreased levels can contribute to depressive symptoms (Craske and Stein, 2016; Heller et al., 2019). Dopamine is also a key neurotransmitter in CNS function, regulating processes including reward, movement, and cognition (Channer et al., 2023).

Vasopressin also influences mood and social behaviors. Research indicates that vasopressin can affect social recognition and bonding, particularly in species like voles, where it plays a role in pair bonding behaviors in a sex-specific manner, with effects typically being stronger in males than in females (Rigney et al., 2023). In humans, vasopressin’s effects on mood may be less pronounced but still significant; its release can be influenced by social interactions and stress levels (Hu et al., 2024). Additionally, vasopressin has been associated with analgesic effects that may indirectly affect mood by modulating pain perception, and these effects have been shown to be more significant in women (Colloca et al., 2016).

#### 3.1.2 Neurological function

The impact of vasoactive agents extends to neurological function as well. NE is involved in modulating alertness and attention through its action on adrenergic receptors in various brain regions. This modulation can enhance cognitive performance under certain conditions but may also lead to neurotoxicity if levels become excessively high or prolonged (Troadec et al., 2001; Álvarez-Diduk and Galano, 2015).

Vasopressin’s role in the CNS includes modulating circadian rhythms and influencing stress responses. The release of vasopressin in the brain can enhance the body’s ability to cope with stressors by promoting adaptive behaviors. Furthermore, the interaction of vasopressin with its receptors in the brain suggests potential neuroprotective effects by reducing neuronal excitability and promoting resistance against stress-induced damage (Corbani et al., 2018).

#### 3.1.3 Potential neurotoxicity

While vasoactive agents have beneficial effects on mood and cognition, there is also potential for neurotoxicity. High levels of catecholamines can lead to neuronal damage due to oxidative stress and excitotoxicity. For instance, excessive catecholamine release during chronic stress can lead to neurotoxic effects on neurons due to oxidative stress and excitotoxicity (Álvarez-Diduk and Galano, 2015). Chronic exposure to elevated NE levels has been associated with neuronal apoptosis and impaired neurogenesis (Jhaveri et al., 2010; Flint et al., 2013). Similarly, excessive vasopressin release can result in adverse effects on neuronal function, particularly when it leads to increased blood pressure and vascular resistance that may compromise cerebral perfusion (Sharshar and Annane, 2008).

Additionally, while vasopressin can enhance social bonding and reduce anxiety under normal conditions, its dysregulation may contribute to maladaptive behaviors or exacerbate anxiety disorders (Hu et al., 2024).

### 3.2 GI effects

#### 3.2.1 Effects on GI motility

Vasoactive agents can alter GI motility through their actions on smooth muscle and neuronal pathways. For instance, catecholamines like NE and EPI primarily act on α and β adrenergic receptors, leading to varied effects on motility. Activation of α1-adrenergic receptors generally promotes smooth muscle contraction, resulting in decreased motility in the GI tract. Conversely, β2-adrenergic receptor activation can lead to relaxation of smooth muscle and increased motility in certain contexts, such as during physical stress when blood flow is redirected to essential organs (Tank and Lee Wong, 2015; Mittal et al., 2017).

#### 3.2.2 Implications for GI blood supply

Vasopressin can activate V1 receptors within the hepato-splanchnic vascular bed, triggering potent vasoconstriction that reduces blood flow in patients with portal hypertension. There was evidence that low to moderate doses of vasopressin resulted in significant reductions in portal blood flow (by 26%–37%) while having no impact on portal or hepatic venous pressures (Bown et al., 2016). Therefore, when treating septic shock, despite achieving hemodynamic stability with vasopressin, there was a notable decrease in mesenteric and portal vein blood flow, which could compromise gut health and function. Whether this reduction in blood flow can lead to ischemia of the GI mucosa, impairing its ability to secrete digestive enzymes and even absorb nutrients, remained unclear (Martikainen et al., 2003).

#### 3.2.3 GI ischemia and bleeding

Strong vasoconstriction caused by NE, phenylephrine, angiotensin II and vasopressin could lead to decreased splanchnic blood flow, resulting in non-occlusive acute mesenteric ischemia. A case series highlighted the potential for high doses of NE to contribute to splanchnic vasoconstriction, leading to non-occlusive mesenteric ischemia in patients with severe acute pancreatitis (Reichling et al., 2020). Similarly, vasopressin, which acts on V1 a receptors to induce vasoconstriction, can also affect splanchnic hemodynamics. In a porcine model of septic shock, a low-dose vasopressin of 0.006 U/kg/h caused a decrease in mesenteric blood flow, resulting in elevated lactate levels and signs of intestinal ischemia (Hiltebrand et al., 2007). Furthermore, the risk of GI bleeding is heightened in patients with increased catecholamine levels due to potential mucosal ischemia and impaired healing responses (Krag et al., 2013). The balance between maintaining adequate perfusion pressure while avoiding excessive vasoconstriction is critical in preventing these complications.

### 3.3 Renal effects

Vasoactive agents, including catecholamines and non-catecholamines, play a significant role in regulating renal blood flow (RBF) and function. Their effects can have profound implications for kidney health, particularly in critically ill patients where the risk of acute kidney injury (AKI) and renal ischemia is heightened.

#### 3.3.1 Effects on RBF

Vasoactive agents influence RBF primarily through their actions on specific receptors located in the renal vasculature. Vasopressin acts predominantly through V1a receptors, which are distributed heterogeneously in the renal circulation. At low doses, vasopressin induces vasoconstriction mainly in the efferent arterioles of the glomeruli, which can theoretically increase glomerular perfusion pressure and enhance glomerular filtration rate (GFR). This mechanism is beneficial in states of hypotension or shock, where maintaining renal perfusion is critical. A study comparing the effects of vasopressin and NE in ovine models of septic AKI demonstrated that NE transiently improved renal function but worsened renal medullary ischemia and hypoxia. In contrast, vasopressin provided a sustained improvement in creatinine clearance without significantly affecting renal medullary perfusion or oxygenation (Okazaki et al., 2020). This suggests that vasopressin may be more beneficial in preserving renal function during septic conditions. Post-hoc analyses from the Vasopressin and Septic Shock Trial (VASST) revealed that patients classified as being at risk for kidney injury had lower rates of progression to more severe forms of AKI when treated with vasopressin compared to NE (Gordon et al., 2010; Lucchese, 2010). Specifically, among patients in the “Risk” category according to RIFLE criteria, those receiving vasopressin showed a significantly reduced need for renal replacement therapy and lower mortality rates (Gordon et al., 2010).

#### 3.3.2 Role of dopamine

Dopamine is known to exert a dose-dependent effect on RBF. At low doses (1–5 μg/kg/min), dopamine primarily stimulates D1-like receptors, leading to renal vasodilation and increased RBF. This effect is attributed to the dilation of afferent arterioles, which enhances GFR and promotes natriuresis (Elkayam et al., 2008; Olivares-Hernández et al., 2021). Low-dose dopamine infusion has been shown to increase mean RBF by approximately 20% in animal models without affecting systemic hemodynamics (Di Giantomasso et al., 2004).

However, the benefits of low-dose dopamine in clinical practice have been challenged. Research indicates that while it may increase RBF in healthy individuals, its efficacy diminishes in patients with AKI or those at risk for renal failure. Studies found that low-dose dopamine worsened renal perfusion in patients with acute renal failure, increasing renal vascular resistance rather than decreasing it (Kellum and Decker, 2001; Lauschke et al., 2006). Therefore, the routine use of low-dose or “renal dose” dopamine for the treatment or prevention of acute renal failure cannot be justified since it has no benefit in either preventing or ameliorating AKI in critically ill patients (Friedrich et al., 2005; Karthik and Lisbon, 2006; Joannidis et al., 2017).

#### 3.3.3 Risk factors for AKI

The use of vasoactive agents carries inherent risks for developing AKI or exacerbating existing renal dysfunction. Key factors include: (1) Vasoconstriction: Renal vasoconstriction induced by vasoactive agents is a well-known phenomenon that may contribute to AKI (Redfors et al., 2011). When vasoactive agents are used to restore systemic blood pressure during shock, they can inadvertently cause renal vasoconstriction, leading to a reduction in RBF, a decline in the GFR, and ultimately, AKI. (2) Hemodynamic Instability: In critically ill patients, fluctuations in blood pressure due to the use of vasoactive agents can contribute to periods of inadequate renal perfusion. Sustained hypotension or rapid changes in vascular resistance can compromise kidney function (Busse and Ostermann, 2019). (3) Underlying Conditions: Patients with conditions such as heart failure, cirrhosis, or sepsis are at higher risk for AKI when treated with vasoactive agents. These conditions often involve complex hemodynamic changes that can exacerbate the effects of these drugs on renal circulation (Ronco et al., 2019). (4) Duration and Dosage: The dosage of vasoactive agents and duration of treatment are critical factors influencing the risk of AKI (Martin et al., 2015). High doses or prolonged use may lead to cumulative adverse effects on kidney function. Current guidelines recommend NE as the first-line agent, but in cases of high NE requirements, the addition of nonadrenergic vasopressors is advised (Venkatesh et al., 2019). This miscellaneous therapies for catecholamine sparing, while physiologically plausible, require careful consideration of patient-specific characteristics to avoid potential adverse effects on renal function.

### 3.4 Endocrine effects

#### 3.4.1 Effects on insulin and glucagon secretion

Vasoactive agents can modulate the secretion of key hormones involved in glucose metabolism, notably insulin and glucagon. Activation of α2-adrenergic receptors in pancreatic β-cells inhibits insulin secretion, which can lead to increased blood glucose levels during stress responses. This is possibly caused by decreasing calcium influx through voltage-dependent calcium channels (Hsu et al., 1991). Conversely, β-adrenergic receptor stimulation enhances insulin secretion during exercise or stress response to facilitate glucose uptake and utilization by muscles, although this effect can be overshadowed by the inhibitory actions of α2 receptors during acute stress (Singh et al., 2018).

Recent studies have highlighted dopamine’s role in regulating pancreatic hormone release (Aslanoglou et al., 2021; Bonifazi et al., 2024). Dopamine acts on both α- and β-cell adrenergic receptors, influencing the secretion of glucagon and insulin (Bonifazi et al., 2024). Notably, dopamine functions as a biased agonist at α2A-adrenergic receptors, preferentially signaling through G protein-mediated pathways to inhibit insulin release (Aslanoglou et al., 2021). This dual action highlights the complexity of hormonal regulation in response to vasoactive agents.

In experiments on mouse islets, it has been shown that vasopressin can significantly amplify glucose-induced insulin release (Szczepanska-Sadowska et al., 2024). Vasopressin also potentiates the stimulatory effects of glucose and ACTH on insulin secretion (Szczepanska-Sadowska et al., 2024). It enhances the release of insulin by glucose in the pancreas via potentiation of paracrine production of glucagon. Glucagon subsequently activates GLP-1 receptors, which play an important role in promoting insulin release (Szczepanska-Sadowska et al., 2024). In addition, stimulation of V1b receptor is essential for the appropriate regulation of the hypothalamic-pituitary-adrenal (HPA) axis during inflammatory stress. Mice deprived of V1b receptor show significantly lower increases in ACTH and corticosterone during acute immune stress, which in turn may affect insulin release (Szczepanska-Sadowska et al., 2024). This indicates that vasopressin, through its regulation of the HPA axis, has also an indirect impact on insulin release.

#### 3.4.2 Implications for metabolic processes

The impact of vasoactive agents extends beyond immediate hormone secretion to broader metabolic processes. Catecholamines stimulate glycogenolysis in the liver through β-adrenergic receptor activation, leading to increased glucose availability during stress (Wang et al., 2024). The role of catecholamines in hepatic glycogenolysis is further mediated by their interaction with the cAMP-protein kinase A (PKA) signaling pathway. Upon activation of β-adrenergic receptors, there is an increase in cAMP levels, which subsequently activates PKA. PKA then phosphorylates glycogen phosphorylase, the enzyme responsible for breaking down glycogen into glucose-1-phosphate, which is eventually converted to glucose (Xu et al., 2014). This pathway highlights the importance of catecholamines in regulating glucose metabolism and ensuring an adequate supply of glucose during stress.

Vasoactive agents also play a significant role in the mobilization of free fatty acids (FFAs) from adipose tissue, which is crucial during periods of stress or fasting when the body requires alternative energy sources. The mechanism through which catecholamines enhance FFA release involves the activation of β-adrenergic receptors, which leads to the phosphorylation of specific proteins that promote lipolysis (Reilly et al., 2020). This process results in the breakdown of triglycerides stored in adipocytes into FFAs and glycerol, which are then released into the bloodstream to be used as energy substrates by various tissues, including the heart and skeletal muscle (Reilly et al., 2020). In addition to their role in FFA mobilization, catecholamines also influence the metabolic fate of these fatty acids. For instance, catecholamines can suppress the re-esterification of FFAs back into triglycerides within adipocytes, thereby favoring their oxidation. This is achieved through the activation of signal transducer and activator of transcription 3 (STAT3), which is phosphorylated upon catecholamine stimulation, promoting FFA oxidation over storage (Reilly et al., 2020).

Research has demonstrated that vasopressin receptor-deficient mice exhibit altered lipid metabolism, characterized by changes in lipid accumulation and metabolism in tissues such as brown adipose tissue and skeletal muscle (Harada et al., 2025). These findings suggest that vasopressin’s regulatory effects on lipid metabolism are mediated through its action on V receptors, highlighting its extensive role in metabolic homeostasis. Further exploration into the molecular interaction between vasopressin and insulin revealed that vasopressin can modulate metabolic processes by influencing insulin secretion and action (Szczepanska-Sadowska et al., 2024). Vasopressin stimulates glycogenolysis and fatty acid synthesis in the liver, while also promoting insulin release from pancreatic cells (Szczepanska-Sadowska et al., 2024). This interaction suggests that vasopressin may play a role in coordinating energy balance and lipid metabolism, potentially impacting conditions such as obesity and diabetes.

### 3.5 Hematological effects on coagulation and platelet function

Vasoactive agents, including catecholamines and non-catecholamines like vasopressin, significantly influence coagulation and platelet function. Their effects can have crucial implications for thromboembolic events, particularly in critically ill patients where the balance between hemostasis and thrombosis is critical (Achaibar and Waldmann, 2015; Neuenfeldt et al., 2021).

Evidence showed that catecholamines enhance *ex vivo* platelet aggregation in healthy donor blood, indicating that they play a role in promoting hemostasis under certain conditions (Matthay et al., 2022). In trauma patients, elevated levels of catecholamines were associated with impaired platelet aggregation and decreased clot strength, suggesting that excessive catecholamine exposure may contribute to a dysfunctional platelet phenotype (Matthay et al., 2022). Catecholamines contribute to platelet aggregation through the stimulation of α2A and β2 adrenergic receptors. This interaction is particularly relevant in the context of acute coronary syndrome (ACS), where catecholamines released during the event can influence platelet reactivity and the efficacy of antiplatelet therapies such as aspirin and clopidogrel (Cuisset et al., 2010). EPI is a special physiological platelet activator that induces platelet aggregation without an initial change in platelet shape. This process involves the production of thromboxane A2, which further enhances platelet aggregation and shape change during the second wave of EPI-induced aggregation (Blockmans et al., 1996). Platelets can accumulate significant amounts of catecholamines, which can affect their activation state and contribute to the overall sympathetic nervous system activity (Zweifler et al., 1990). Moreover, the uptake and retention of catecholamines by platelets are influenced by the concentration and duration of exposure to these hormones. Catecholamines also stimulate the release of coagulation factors such as factor VIII (FVIII) and von Willebrand factor (vWF) from endothelial cells (Han et al., 2017). Thus, EPI was once used to treat von Willebrand’s disease, the most common inherited bleeding disorder worldwide (Rickles et al., 1976). This action contributes to a hypercoagulable state, particularly during acute stress responses when catecholamine levels are elevated. The release of these factors enhances clot formation but can also increase the risk of thrombosis if not properly regulated.

Vasopressin has been shown to have direct procoagulant effects through its action on V1a receptors located on vascular smooth muscle and platelets (Hasan et al., 2006). Activation of these receptors leads to increased platelet aggregation and the release of vWF, enhancing the ability of platelets to adhere to the damaged endothelium (Casonato et al., 2015). In addition, extra-renal V2 receptors activation induces the release of coagulation factors (Demiselle et al., 2020). Desmopressin, a synthetic analogue of vasopressin, has been widely recognized for its efficacy as a hemostatic agent in the management of inherited bleeding disorders (Mohinani et al., 2023). This compound is particularly effective in conditions such as mild hemophilia A and von Willebrand disease, where it functions by increasing the levels of coagulation FVIII and vWF in the circulation (Mohinani et al., 2023). The mechanism of action involves the stimulation of extrarenal V2-receptors, which leads to the release of these factors from endothelial cells, thereby enhancing hemostasis (Mohinani et al., 2023). Moreover, desmopressin has been demonstrated to be safe in managing bleeding complications during pregnancy in women with congenital bleeding disorders (Al Arashi et al., 2024).

## 4 Clinical implications in patient management

### 4.1 Importance of monitoring non-cardiovascular effects

While vasoactive agents are critical for managing hemodynamic instability in critically ill patients, their use carries significant risks for adverse effects on non-cardiovascular systems. These effects include renal impairment, GI ischemia, neurological disturbances, hematological complications, and endocrine dysregulation. Awareness of these potential complications is essential for clinicians to optimize treatment strategies and minimize risks associated with vasoactive therapy.

Thus, continuous monitoring allows early detection of non-cardiovascular adverse effects. For instance, observing changes in urine output can signal renal impairment due to reduced renal perfusion from vasopressor therapy. Similarly, monitoring GI symptoms can help detect potential ischemia or bleeding early, allowing for timely intervention. Each patient’s response to vasoactive agents can vary significantly based on underlying health conditions, comorbidities, and the severity of their illness. Regular assessment enables healthcare providers to tailor treatment plans according to individual patient needs, adjusting dosages or switching agents as necessary to minimize adverse effects.

By actively monitoring non-cardiovascular effects, healthcare providers can implement preventive measures that may improve overall patient outcomes. For example, recognizing signs of hypercoagulable state early can lead to prompt adjustments in therapy or supportive care strategies that mitigate complications such as deep vein thrombosis. Understanding the potential for adverse effects allows for better risk stratification among patients receiving vasoactive therapy. This information is crucial in prioritizing monitoring efforts and determining which patients may require more intensive observation based on their risk profiles.

### 4.2 Strategies to mitigate risks associated with non-cardiovascular effects

Firstly, establishing standardized protocols for monitoring vital signs, fluid balance, renal function (e.g., serum creatinine), and GI symptoms can help healthcare teams identify potential issues early. Implementing checklists or electronic health record alerts can facilitate adherence to these protocols. Secondly, engaging a multidisciplinary team—including intensivists, pharmacists, dietitians, and nursing staff—can enhance the management of patients receiving vasoactive agents. Collaborative discussions regarding medication management and potential side effects can lead to more comprehensive care strategies. Thirdly, providing education for healthcare professionals about the potential non-cardiovascular effects of vasoactive agents is essential. Training programs should emphasize recognizing early signs of complications and understanding the pharmacological mechanisms underlying these effects. Fourthly, careful fluid management is crucial in mitigating renal complications associated with vasoactive agents. Employing dynamic assessments of fluid responsiveness (e.g., using ultrasound or other hemodynamic monitoring techniques) can guide fluid resuscitation efforts while avoiding volume overload. Lastly, engaging patients in their care by discussing potential side effects and encouraging them to report any unusual symptoms can enhance monitoring efforts. Educating patients about the importance of reporting changes in their condition fosters a collaborative approach to care.

Of note, some emerging technologies or alternative therapies could be developed to monitor and mitigate the non-cardiovascular effects of vasoactive agents. Combined usage of multiple vasoactive agents with different mechanisms, also termed ‘broad-spectrum vasopressors’, can be an effective strategy to mitigate non-cardiovascular side effects (Wieruszewski and Khanna, 2022). This multimodal approach leverages the distinct pathways and actions of various agents to achieve therapeutic goals while minimizing adverse effects.

AI-driven algorithms have increasingly been applied in healthcare settings to predict and prevent adverse effects associated with various medications. These algorithms leverage machine learning techniques to analyze large datasets, identifying patterns and risk factors that may not be immediately apparent to clinicians (Classen et al., 2023; Litvinova et al., 2024). By doing so, they can provide early warnings and suggest interventions that could mitigate potential adverse effects, thereby enhancing patient safety and improving clinical outcomes (Litvinova et al., 2024). Furthermore, the use of AI in pharmacovigilance has been explored to automate signal detection and manage adverse drug events (Wadhwa et al., 2021). This approach involves data mining techniques to identify potential signals from various sources, including clinical trials and post-marketing data. By automating the detection of adverse events, AI-driven systems can provide timely alerts and facilitate the prevention of adverse effects associated with vasoactive agents, thereby improving patient safety and healthcare outcomes.

Additionally, the use of CRISPR-Cas9 technology in precision gene editing offers a novel approach to understanding and potentially mitigating the non-cardiovascular effects of vasoactive agents. By enabling precise modifications at the genetic level, CRISPR can help elucidate the pathways through which these agents exert their effects, paving the way for more targeted therapies that minimize unintended consequences (Legere and Hinson, 2024).

## 5 Future directions

Despite the widespread use of vasoactive agents, significant knowledge gaps exist regarding their non-cardiovascular effects. There is a need for comprehensive studies examining how vasoactive agents affect renal function, GI health, neurological status, and coagulation pathways. For example, elucidating how catecholamines influence neurotransmitter release or how vasopressin affects renal tubular function could lead to better therapeutic strategies and minimize adverse outcomes. Similarly, the impact of these agents on GI ischemia and bleeding requires more targeted research to develop effective monitoring and intervention strategies. There is also a lack of long-term studies assessing the chronic effects of vasoactive agents on non-cardiovascular systems. Most existing research focuses on short-term outcomes, which may not capture the full spectrum of potential adverse effects that could arise from prolonged exposure to these medications.

Therefore, addressing the non-cardiovascular side effects of vasoactive agents requires a multifaceted approach involving further research into their mechanisms and long-term impacts, as well as innovative strategies for developing novel therapies.

## 6 Conclusion

Vasoactive drugs are essential for managing critical conditions like shock but can have significant non-cardiovascular effects that require attention. This review examines their impact on renal function, GI health, neurological status, and coagulation pathways. These non-cardiovascular effects require careful monitoring and innovative research to enhance patient safety and outcomes.
